# Harnessing routinely collected data for the evaluation of early years interventions: insights from a scoping review of evaluation studies

**DOI:** 10.23889/ijpds.v6i1.3378

**Published:** 2026-09-17

**Authors:** Kate E. Mooney, Hollie Henderson, Kelly Hollingsworth, Sarah L Blower, Jenna Graham, Rina Davidson, Leena Ali, Farwa Batool, Dea Nielsen

**Affiliations:** 1 Department of Health Sciences, University of York, York, YO10 5DD, United Kingdom; 2 Born in Bradford, Bradford Institute for Health Research, Temple Bank House, Bradford Royal Infirmary, Duckworth Lane, Bradford, BD9 6RJ, United Kingdom; 3 A Fairer Start, Nesta, 58 Victoria Embankment, London, EC4Y 0DS, United Kingdom

**Keywords:** routine data for evaluation, early intervention, early child development, outcomes

## Abstract

**Introduction:**

Routinely collected data (RCD) describes data about individuals that is documented routinely in practice. Evaluation of early years interventions is imperative for improving child health and development, and utilisation of RCD for research could overcome limitations with traditional study designs, such as participant burden, attrition and disappointment bias. A systematic understanding of if (and how) RCD is being used to evaluate early years interventions is lacking.

**Objectives:**

To scope the literature on how RCD is being used to evaluate the effectiveness of early years interventions being delivered in the UK.

**Methods:**

The study protocol was registered online (osf.io/cug36). Included studies were interventions for expectant parents and parents of children aged 0-5 years, with a quantitative measure obtained from RCD as an outcome. The study scope was limited to the UK, from January 2000 to November 2024. Completed studies and study protocols were eligible, encompassing grey literature and peer reviewed sources. Medline, Psycinfo and Embase databases were searched via Ovid.

**Results:**

32 unique studies published 2009-2024 had used or were planning to use RCD as an outcome in an evaluation of an early years intervention. Most measured more than one outcome, and the most common were birth outcomes and child education. Many studies noted limitations of RCD, particularly being limited by measures available, and fewer noted strengths.

**Conclusion:**

Whilst RCD expands evaluation opportunities for measuring multiple relevant parent and child outcomes simultaneously, researchers are limited by what measures are available in RCD. We make specific recommendations to improve the use of RCD in early years evaluations. With careful consideration, RCD can provide useful insights regarding the impacts of early years interventions. This scoping review reveals an emerging methodology that may have increasing importance for evaluating early years policies and interventions, but may currently be constrained by data availability and quality.

## Introduction

Providing all children with the best start in life is a high priority recommendation highlighted by UK Government policies, showing a clear political commitment to early years support and investment [1]. Early intervention delivered to children and their caregivers during pregnancy and in the first five years of their child’s life could prevent the onset of poor parental and child outcomes, and mitigate the potential personal, familial, and societal costs of longer-term negative outcomes [2]. Early interventions can impact a range of caregiver and child outcomes, from physical health (e.g. birth outcomes, breastfeeding), wellbeing (i.e. maternal mental health, child socioemotional development), as well as developmental, cognitive and linguistic skills at the level of the child (i.e. early educational measures). Despite the potential benefits of intervening early being well documented [3], there remains a lack of robust evidence for the short and long-term effectiveness of interventions for parents of children in the critical early years of life [2, 4].

This lack of robust evidence for such interventions may be due to the numerous challenges with evaluating them. Where individual services to support parents exist within local authorities, service providers may be reluctant to engage in randomised studies, as they prioritise providing support to those who need it most. Service providers may understandably be reluctant to use a randomised design because it could mean denying a potentially beneficial intervention to a family in need [5–7]. Randomised studies that evaluate such interventions may also suffer from limited representativeness and generalisability, as parents who are at the point of ‘readiness to change’ are unlikely to agree to take part in a study in which they might be randomised not to get help, resulting in recruitment bias [8]. Furthermore, such studies are often susceptible to recruitment challenges, particularly relating to a high number of participants being lost to follow-up [9], especially when exploring long-term effects of the intervention. For example, even with multiple incentives to support low-income mothers to remain in a parenting intervention, 41% of mothers were lost to follow-up [10].

A potential solution to some of these challenges is to use routinely collected data (RCD), which describes data that are collected about individuals when they interact with public services, such as health, education and social care services, as part of their service delivery and practice [11, 12]. RCD can be used in studies examining the impact of factors such as early interventions, on parent and child outcomes [13, 14]. Hence, a solution to the issues with evaluating early years interventions is to instead utilise the data which are already, in theory, universally and routinely collected in parent and child records, overcoming limitations with recruitment and generalisability. RCD at a population level can also enhance generalisability and allow better comparison to similar populations [7]. RCD enables both short and long-term outcomes to be obtained, which reduces the participant burden of extra data collection, and potentially reduces attrition and disappointment bias. This can be applied for both randomised and non-randomised designs, which means follow-up periods can be extended for randomised studies, and allows the realisation of observational studies of interventions. The use of non-randomised designs also reduces the ethical concern for the service regarding randomisation; as no participant who desires the intervention will later be randomised to a control group. However, it is also important to be aware of the limitations of non-randomised studies for making causal claims about the impacts of interventions [15].

It is important to note that since RCD is not designed for research purposes, its use for evaluating early years interventions must be considered with limitations in mind. One limitation is that the collection of RCD varies within the nations of the UK. For instance, whilst the Ages and Stages Questionnaire (ASQ) is a routinely used measure of child development as part of the Healthy Child Programme in England [16], the Schedule of Growing Skills (SOGS) is used in Wales [17]. These differences limit the scope for evaluations across multiple nations. Previously noted concerns about RCD include the relevance of the RCD to the specific intervention, and the completeness and quality of the data itself [18–20]. Regarding the relevance of the RCD, the effects of specific programmes may risk going unobserved if the routinely collected outcome lacks sensitivity to specific changes[19, 20]. A systematic review of the challenges and strategies for using RCD in research highlighted residual confounding, misdiagnosis, misclassification, and missing data as concerns [21].

New initiatives are facilitating access to routine data at both local [22, 23] and national levels [12, 24, 25] for research purposes. For instance, RCD has been used to create Scotland’s first administrative child cohort, linking records for over 198,483 mother-child pairs [24], and England’s Education and Child Health Insights from Linked Data Mother-Baby (ECHILD-MB) cohort was created by linking 13.6 million baby records to mothers [25]. These datasets can potentially be used to evaluate early years interventions, where information about intervention exposure has been routinely collected, or where population wide policies or interventions can be evaluated via natural experiments [15].

**Table 1:** Participant, Concept, Context (PCC) Framework and eligibility criteria

**Inclusion criteria**	
**Participants**	Participants can be parents or children: If the participant is a parent/guardian, participants included in the study must either be pregnant (or have a pregnant partner), or have a child aged between 0 and 5 years old at the time the intervention was delivered. If the participant is a child, the child must be aged between 0 and 5 years old at the time the intervention was delivered.
**Concept**	Any interventional study design that reports quantitative information: e.g. quasi-experimental, before and after, Randomised Control Trials, etc. Intervention was delivered when the parent was pregnant, and/or, when child was aged 0-5 years old. Study must include use of a quantitative outcome measure obtained from routinely collected data at any time in the parent/child’s life. Studies can be planned, ongoing or completed: Planned studies may be protocols or registrations. Completed studies may be reports/published literature.
**Context**	Intervention was delivered in the UK. Any study or protocol published/reported since the year 2000. The intervention is social and/or behavioural in nature, and can be delivered in any context and be broad in its scope (e.g. can be a national or local policy, or can be commissioned and delivered locally through an organisation or the voluntary sector).

### Present study and rationale

Evidence for successful early years interventions is lacking, despite a renewed policy interest in providing all children with the best start in life [1, 3]. Despite its limitations, utilising RCD as an outcome could overcome several evaluation challenges. Hence, a systematic understanding of what components of RCD are being, or have been, used to evaluate early years interventions and/or policies, is crucial for planning of future evaluations. This may enable identification of areas where improvements to existing RCD infrastructures may be required, and identification of RCD that is being underused. Given the geographical variability of what and how data are collected between countries, it is important to consider these questions within the specific area of interest, in this case the United Kingdom.

This scoping review therefore aims to gain an overview of both planned and completed studies that have used an outcome measure obtained from RCD in evaluations of early years interventions (delivered either during pregnancy, or up child age 5 years old). Our focus is on universal and targeted interventions with a social and behavioural component, rather than interventions or products to treat specific conditions. The scoping review question is:

1. ***How is routine data being used to evaluate interventions delivered in the early years in the UK?***

## Methods

A preliminary search of PROSPERO, the Cochrane Database of Systematic Reviews and JBI Evidence Synthesis was conducted and no current or underway systematic reviews or scoping reviews on the topic were identified. This scoping review was conducted in accordance with the JBI methodology for scoping reviews [26], and reported using the PRISMA Extension for Scoping Reviews (PRISMA-ScR) [27] (Appendix 1). The protocol was registered at https://osf.io/cug36/.

### Eligibility criteria

The ‘Population, Concept, Context’ framework was used to describe the eligible studies. Broadly, we focused on studies that included interventions delivered to caregivers with a 0-5-year-old child, and/or interventions delivered directly to children aged 0-5 years. The concept of the interventions was broad and could be any kind of intervention and/or policy that was delivered when parents were pregnant, or when the child was aged 0-5 years old. The context was UK only, as the purpose was to inform the use of routine data use in intervention evaluations within UK contexts. Studies published since 1st January 2000 were included, to ensure that any results are relevant to modern data systems.

Table 1 describes the framework and eligibility criteria. A more detailed table with exclusion criteria for guiding the screening process was published with the study protocol (see osf.io/cug36/). At abstract screening, we included any studies that: (a) were social and/or behavioural interventions and used a quantitative design, (b) were about parents/children (aged 0-5 years old) and (c) were in the UK, or it was not clear where the study took place. Clinical Trials of Investigational Medicinal Products (CTIMPs) were screened out at both abstract and full text screening stages, meaning most remaining intervention studies were social and/or behavioural. If any uncertainty arose regarding the eligibility of an intervention, two authors (KEM and DN) discussed and agreed on these. If both a study protocol and published results were available, we retained only the study results paper due to it containing more relevant information to our scoping review question. We hand searched for published studies resulting from protocols and instead included these where available, hence any protocol studies represent those not yet published.

### Types of sources

Peer reviewed studies and grey literature (including reports, websites, and conference abstracts) were both eligible for inclusion. We included studies at any stage from study protocols, interim findings, to study reports of outcomes.

### Published literature search strategy

The full search is provided in Appendix 2. The words associated with the population, concept and context of the review were used to guide the search terms. The National Institute for Health and Care Excellence (NICE) filters were used to identify studies conducted within the United Kingdom [28]. Medline, Psycinfo and Embase were searched via Ovid for peer reviewed literature published between January 2000 and 13th November 2024.

### Grey literature search strategy

Grey literature searches took place in November 2024. We targeted specific websites relevant to our topics, namely National Institute for Health Research (NIHR), Education Endowment Foundation (EEF), Early Intervention Foundation, National Foundation for Education Research (NFER), ISRCTN (https://www.isrctn.com/), Clinicaltrials.gov, GOV.UK(https://www.gov.uk/search/all), and GOV.wales (https://www.gov.wales/). Records of websites searched, number of potentially relevant items, and number scanned were kept in Microsoft Excel [29].

### Evidence selection

Titles and abstracts were uploaded to Covidence for screening. A piloting process took place with 100 studies, and agreement between all reviewers was reviewed. After piloting, amendments were made to the inclusion criteria to improve the clarity of the included studies. All titles and abstracts were screened by two reviewers according to the criteria described above for abstract screening. Cohen’s Kappa scores were reviewed regularly as a team, since lower than desired agreement (<0.60) occurred between some pairs of reviewers. Regular meetings and reviews took place with the whole team to resolve disagreements, and Cohen’s Kappas improved. After abstract screening, the full texts were screened and agreed by two reviewers (DN or KEM).

### Data extraction

Data was extracted from eligible papers by three reviewers (RD, JG, KH). A proportion of 20% of selected articles was second checked by KEM to ensure accuracy. Data included specific details about the participants, concept, context, study methods and key detail regarding the outcome measurement, source of RCD, and data availability statements. Discussions sections from the included studies were examined by KEM to extract a list of specific strengths and limitations associated with using RCD that the study authors mentioned.

### Data analysis and presentation

A full list of studies and characteristics is provided in Appendix 3. The included studies were summarised in terms of their study types, designs, intervention types, and outcomes used including the type of outcome, timing, and whether the outcome was reported at the parent or child level. Due to the primary interest of the study being in the outcome measures that were obtained from RCD, we extracted data on the specific outcome measures used by individual studies. KEM categorised these into broader outcome measures so that they could be summarised, and co-authors reviewed these. The resultant categories and examples of what specific measures they included were:

- Birth outcomes, relating to birth experience or immediate post birth experiences such as mode of delivery, or length of stay in hospital post birth
- Service implementation, relating to how/if a service was implemented, such as whether a specific appointment took place e.g. child health reviews by GPs
- Smoking status, relating to whether a mother was recorded as smoking
- Maternal healthcare use, relating to a mother’s healthcare use e.g. hospital stays
- Child healthcare use: as above, but for the child
- Maternal reproductivity, relating to subsequent maternal pregnancies
- Maternal health or survival, relating to any maternal health outcome e.g. diabetes, or maternal survival/death
- Child health or survival: as above, but relating to the child
- Maternal mental health, relating to any mental health outcome e.g. identification of poor mental health via GP records
- Child educational attainment and/or development
- Child abuse, neglect, maltreatment, and/or protection
- Breastfeeding, relating to any record of breastfeeding and/or not breastfeeding
- Domestic violence, relating to any incidents recorded for the mother
- Child weight
- Maternal educational attainment

Data were charted by these study characteristics, and bar graphs created where they provided additional insights. Data was also extracted by KEM from individual studies’ strengths and limitations sections, and summarised according to broad themes, which were reviewed by co-authors.

## Results

### Search results

**Figure 1:**
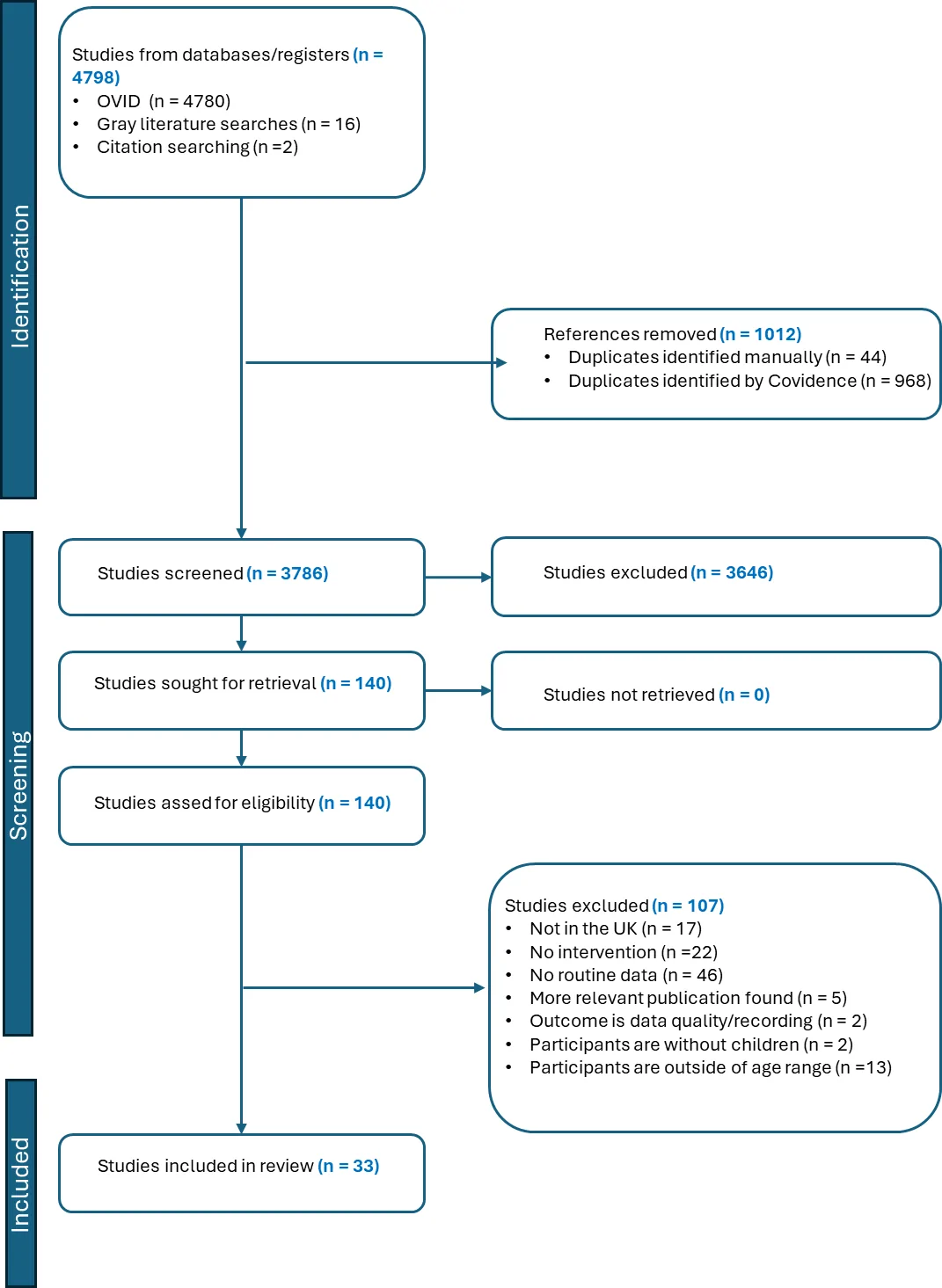
PRISMA flow diagram for scoping review process

Figure 1 presents the flow of included studies. There were two studies using the same data source, but they contained different periods of follow-up routine data, hence, we retained these two studies in the review. The studies are (1) a protocol of a randomised study to evaluate the Family Nurse Partnership [30] and the results from the routine data follow-up from that study [31].

### Study characteristics

**Table 2:** Included studies characteristics

**Characteristic**	**N (%)**
**Study type (n = 33 studies)**	
Protocol	8 (24)
Published study	25 (76)
	
**Study design (n = 32 unique studies)**	
Observational studies (inc. natural/quasi-experimental)	23 (72)
Randomised trial	9 (28)
	
**Geographic location of study (n = 32 unique studies)**	
England	
*National level*	8 (24)
*Specific place in England*	12 (38)
Scotland	8 (24)
Wales	2 (6)
Multiple nations	2 (6)
	
**Intervention focus (n = 32 unique studies)**	
Antenatal programme	9 (29)
Service/policy change	8 (25)
Smoking cessation	3 (10)
Breastfeeding	3 (10)
Parenting programme	3 (10)
Voucher programme	2 (6)
COVID-19	2 (6)
Weight management	2 (6)
	
**Outcome data (n = 31 unique studies)**	
Parent	11 (34)
Child	10 (32)
Both	11 (34)
	
**Time period of latest outcome data (n = 31 unique studies)**	
Pregnancy	2 (6)
Birth	7 (22)
Post birth	10 (32)
	
Up to child age:	
*1 year*	3 (3)
*3 years*	3 (9)
*4 years*	1 (3)
*5 years*	4 (13)
*6 years*	1 (3)
*7 years*	2 (6)

Table 2 summarises key characteristics of the included studies, and the extracted data for all studies is provided in Appendix 3. This information is used in the narrative synthesis below.

### Narrative description

#### Description of studies

Out of 33 studies, 25 were published studies with results (76%), and 8 were study protocols (24%). Out of 32 unique studies, most (n = 23, 72%) were observational (ie. non-randomised interventional studies). The remainder were randomised control trials (n = 9, 28%) [30, 32–40].

**Figure 2:**
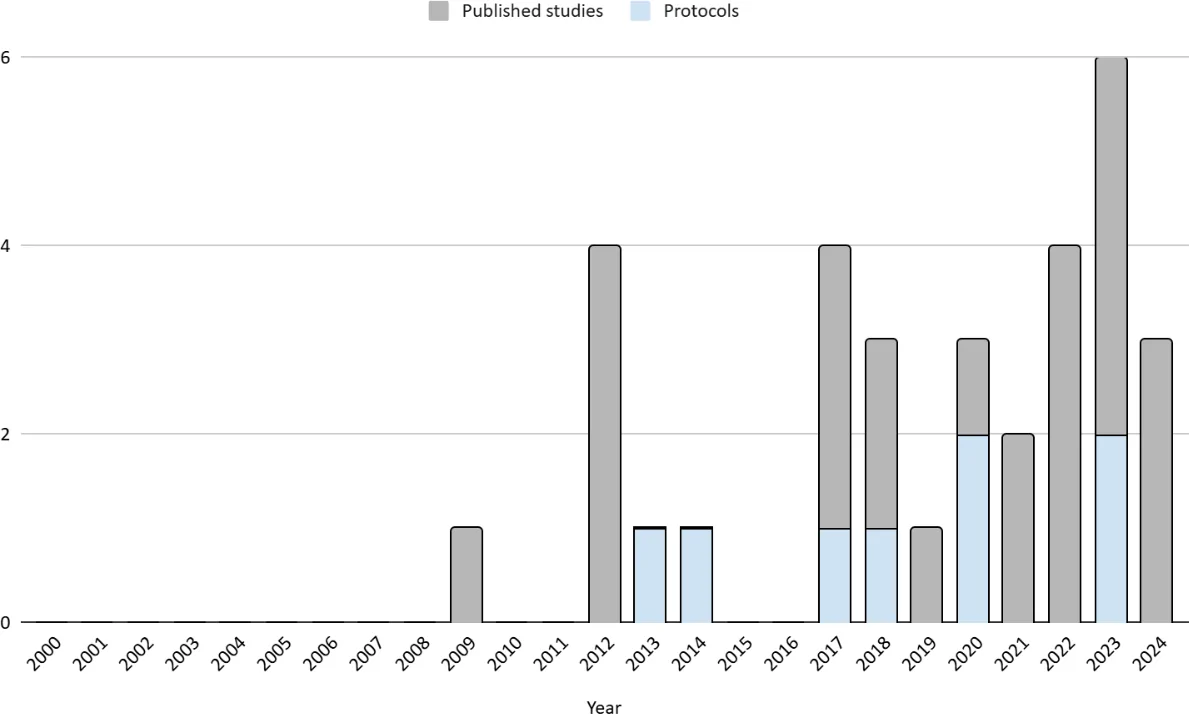
Number of studies published per year

Figure 2 demonstrates that the first eligible study was published in 2009. Generally, the number of published studies eligible for our review has increased with time, with the most being six studies in 2023, which included two protocols and four published studies.

#### Intervention type

Most (n = 9, 28%) were perinatal interventions, meaning that they were broad perinatal support interventions that started antenatally, with some continuing postnatally. This included programmes such as the Family Nurse Partnership [30, 37, 41, 42], “Pregnancy Circles” [34, 43], and “Baby Steps” [44]. The second most common category were service and/or policy evaluations (n = 8, 25%). This included a broad remit of intervention types ranging from, for example, the availability of Community Perinatal Mental Health teams in local areas [45], to an evaluation of the impact of a Growth Assessment Protocol implemented in maternity [46]. The other categories were smoking cessation in pregnancy (n = 3, 9%), breastfeeding support programmes (n = 3, 9%), parenting programmes (n = 3, 9%), voucher schemes (n = 2, 6%), weight programmes (n = 2, 6%), and the effect of COVID-19 policies (n = 2, 6%).

#### Routinely collected outcomes

**Figure 3:**
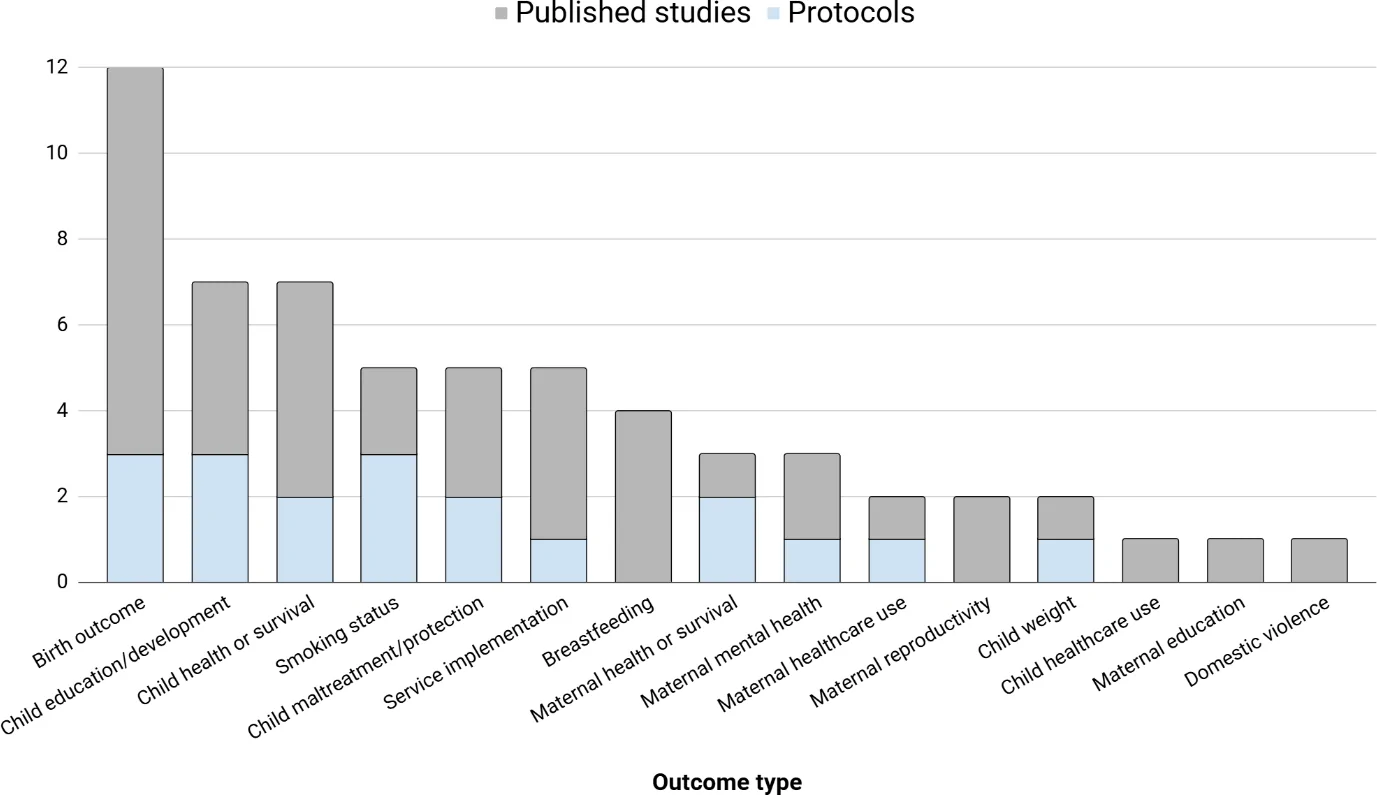
Outcome types measured in all studies, separated by protocol and published studies

*Note: studies may have measured >1 outcome, so double counting of studies occurs.*

There was an even spread of studies which focused on only parent outcomes (n = 10, 32%), only child outcomes (n = 11, 34%), and both parent and child outcomes (n = 11, 34%). Figure 3 shows that we categorised outcomes into 14 broad categories. More than half of the studies measured more than one outcome type (n = 20, 63%), with the most being six different outcome types in one study [41]. The most common category was birth outcomes (n = 12, 38%), which included a broad range such as gestational age, birth type, and stillbirths. These studies mentioned use of maternity electronic patient records [47], Hospital Episode Statistics (HES) [48], and routinely collected NHS data [49]. The next most common was child education and/or development outcomes (n = 7, 22%), with all but one of these studies mentioning use of the Early Years Foundation Stage Profile (EYFSP) linked via the National Pupil Database 31, 38, 41, 42, 50–52]. The one study which did not use the EYFSP was a study evaluating the impact of the universal health visiting pathway in Scotland on child developmental concerns at age 27-30 months [52].

The joint next most common was child health or survival (n = 7, 22%), including child outcomes at birth including gestational age [53, 54], child survival [46, 55], and specific child health needs such as respiratory support [46], all via electronic patient records, NHS digital, or NHS National Services Scotland. The next most common was smoking status (n = 5, 16%), recorded in maternity records during pregnancy [49, 52, 56–58]. The joint next most common was child maltreatment (n = 5, 16%), with one study linking this via child protection status in administrative data [59], one using social care data [41], one using local authority child protection register [60], and one ascertaining this via Child in Need (CIN) status via the National Pupil Database [42]. The joint next most common outcome was service implementation (n = 5, 16%), which related to the proportion of women attending antenatal bookings [34, 43], child health reviews conducted by GPs [61], and service use of a community perinatal mental health team [45].

We categorised studies into the timing of their most recently routinely collected outcome. The most common period was up to 3 months post birth (n = 10, 31%). The outcomes included in this period included length of stay in hospital [46, 48] and readmission [48] (both categorised as birth outcomes), breastfeeding outcomes [32, 33, 40, 54], and survival for the parent and/or baby [46, 55]. The next most common follow-up period was at birth (n = 7, 22%). Fewer studies examined outcomes beyond 5 years (n = 7, 22%).

We extracted data availability statements from the published studies with results. We found that most studies (n = 16, 52%) reported data availability and/or sharing statements, or reported detailed descriptions of datasets if they were funder reports, which typically allow for a larger word count than journal articles [31, 41, 49, 54]. The studies which did not include data availability statements (e.g. [33, 57, 61]) tended to have been published earlier than studies which did (e.g. [55, 62, 63]).

#### Limitations and strengths noted in studies

Appendix 3 presents the list of strengths and limitations per study, and we summarise the findings here. Most of the studies (n = 21, 64% of 33 studies total) mentioned one or more specific limitations of using RCD. A common limitation noted by nine studies was that the choice of outcome measurement was limited by what was available in routine data [31, 44, 50, 51, 53, 57–59, 61]. Two studies noted that measures of maternal-child interaction and/or bonding would be beneficial [41, 51]. Another limitation was the high level of missing data [45, 49, 60, 64]. Other common limitations noted were the length of time to gain permissions to access and receive data [41, 50], and concerns in the accuracy of the data [32, 45, 62].

Fewer studies mentioned strengths of using RCD (n = 9, 28% of 33 studies total). Several studies noted the increased sample size achieved through using RCD, noting whole population coverage [32, 34, 47, 55], low missing data, low loss to follow-up [33], and improved power and/or greater precision in estimates [45, 49]. Two studies noted that using RCD is cost-effective, highlighting the low burden for participants [42, 49]. Two studies noted the relevance of the outcomes to clinical practice and/or policy making decisions [47, 51]. One study noted the minimisation of bias in self-reporting, and the potential to follow-up participants over time [42].

## Discussion

### Summary of studies

We identified a total of 32 unique studies published between 2000-2024 that had used or were planning to use RCD as an outcome to evaluate an early years intervention. The majority were publications with results (76%), with the remainder being study protocols (24%). The studies were predominantly based in England (64%), followed by Scotland (24%), Wales (6%), or multiple nations (6%). The few studies using data from >1 nation may suggest a challenge in harmonising RCD across the UK’s devolved nations, since these nations operate separate early years services and hence separate data collection systems [16, 17]. The first eligible study was published in 2009. The number of eligible studies has increased over time, with the highest number being six in 2023. This could reflect both a developing field due to an increased policy demand for effective early years interventions [1, 65], and an increase in data linkage research and capabilities [13, 25].

Most studies employed observational designs (n = 23, 72%). Observational studies were likely most frequent due to the nature of RCD, which is that it is primarily collected for purposes other than research [66, 67]. RCTs typically represent funded studies which do not usually need to rely upon RCD. The most common type of intervention were perinatal programmes (29%). This may reflect the availability of RCD in maternity records, meaning these programmes are readily evaluable using RCD on birth related outcomes.

### Outcomes in routinely collected data

The common use of both parent and child outcomes demonstrates the benefits of using RCD, as it can assess the dual impact often targeted by complex interventions delivered in the early years (e.g., the Family Nurse Partnership, which aims to improve both maternal health/life chances and child outcomes [31, 41]). Evaluations that measure both categories are best positioned to capture the full scope of an intervention’s potential effectiveness.

Most studies measured more than one outcome type via RCD. This highlights a strength of using RCD, as it has the capacity for comprehensive and multi-purpose evaluation with no additional data collection burden for participants or researchers. This aggregation may allow evaluations to move beyond a single "primary" outcome, providing a more comprehensive understanding of an intervention’s effects across various domains. As noted by several of our included studies, use of RCD is highly cost-effective and low burden compared to collecting multiple different outcomes through bespoke surveys [42, 49].

The most common outcomes were birth outcomes, child education/development, and child health. The commonality of birth outcomes may, again, reflect the availability of RCD in maternity records to be used in such evaluations. These birth outcome data indicators may be recorded with higher completeness than other RCD, because they are embedded into midwifery practice, and essential for clinical decision-making. This is apparent in previous research which has assessed the potential utility of RCD by maternity services for research purposes, finding the completeness of this data to be acceptable [68]. These indicators therefore may serve as reliable, early indicators of intervention success, particularly for antenatal programs. The frequent use of the EYFSP to measure child development is likely due to it being a mandated assessment in England, meaning it has high coverage for this population at school entry [69]. Use of the EYFSP may allow researchers to assess the long-term developmental impact of perinatal interventions, a key advantage over short-term studies. This would also reflect a key policy interest, with the UK Government target for 75% of children to have a Good Level of Development on the EYFSP by 2028 [1].

The routinely collected ASQ®-3 by health visitors at the 2-2 1/2 year review completed in England’s Healthy Child Programme [16, 70] was indicated to be used by only one study, which was in Scotland [52]. The ASQ is a broad measure of a child’s early development and could be a targeted outcome for many interventions, and this suggests that this potential measure may be being underutilised, particularly in England. However, whilst the ASQ should be universally completed as a mandated assessment in England [16, 70], recent studies have indicated variation in the completeness of the ASQ [71], which may explain its underuse as an outcome in evaluations. All nations within the UK deliver universal services with routine assessments of early child development [72–74], however, nations appear to differ in when and how they measure child development, meaning it would be challenging to harmonise these data and evaluate the impact of UK wide policies on early child development. The two studies included in this review which included >1 UK nation focused on birth outcomes and child survival [55, 75], potentially reflecting this.

Whilst many studies targeted the same outcomes, they differed in their descriptions of their data source. For instance, several studies utilised routine maternity data, however, it was challenging to determine if they had used the same data sources e.g. "maternity electronic patient records," and "routinely collected NHS data". Studies also differed in whether they included data availability statements or not, with more recent studies being more likely to include them. Studies may have used the same underlying data source, or distinct datasets, and the variation in language and inconsistent use of data availability statements, particularly in older studies, makes this difficult to ascertain. The lack of standardisation in describing routine data sources presents a barrier for researchers seeking to use RCD to evaluate early years interventions.

The timing of the outcome varied, with the most common being up to 3 months post birth (32%), at birth (23%), and at child age 5 years (13%). This indicates that most studies used RCD in the very earliest years of children’s lives, and fewer studies successfully leveraged RCD for medium-to-long-term follow-up (with only 3 studies examining outcomes post 5 years old). A main benefit of RCD is the possibility for long-term follow-up with minimal participant burden. It is possible that as access to RCD improves, more studies linking long-term RCD to evaluations will emerge.

### Strengths and limitations noted in studies

Most studies noted specific limitations in using RCD in their studies (64% of 33), with the most frequent being the limited choice of outcome measurement based on what was available in the RCD. This limitation highlights that crucial, but more nuanced, proximal outcomes (e.g., parental confidence, quality of parent-child interaction) that might typically be collected may be missed in a study relying upon RCD [19, 20, 41, 51]. This limitation will be compounded for quasi-experimental studies relying on RCD, as they will be unable to control for confounding by multiple risk factors which are not routinely measured – a limitation noted in one of our included studies [41]. Further, the measures collected in RCD may not be the most valid and reliable measures of specific outcomes. This could lead to an incomplete picture of an intervention’s effect, particularly if the mechanism of change is not directly measurable in RCD. Political interest in using population based data for evidence based decisions is crucial for developing new databases and data collection systems [7], hence the current Government Best Start policy [1] highlights the timeliness of this review.

Authors also noted concerns regarding accuracy of the data, and this should be investigated by researchers, for instance via studies which examine the implementation of RCD in local practice [76]. These limitations reflect that RCD are not designed for research purposes, and its use should always be considered with its limitations in mind [18, 21].

Fewer studies noted strengths related to using RCD (30% of 33 studies), which most frequently included an increased sample size and whole population coverage, and low missing data or low loss to follow-up. RCD may address previously noted challenges with evaluating early years interventions, including that such interventions are subject to a high attrition rate [8, 10]. Altogether, the limitations and strengths highlighted a critical trade-off when using RCD to evaluate interventions: whilst the use of RCD addresses some challenges with evaluating early years interventions, researchers are also limited by what was available in RCD and the quality of these data.

### Recommendations

1. **Further research into which outcomes policymakers should integrate into RCD.** RCD currently misses key outcomes for early years interventions [19]. Two of our included studies noted that parent-child interaction measures would be beneficial to understand impacts of the studies interventions [41, 51], and this, together with secure attachment, regular bedtimes, and parenting confidence have all been noted as appropriate outcomes of early years interventions which are not currently measured in RCD [19]. The challenges of ascertaining perinatal mental health via RCD have been highlighted in other studies [77]. Further research with both clinicians and researchers is needed to explore which outcome measures should be prioritised, to ensure measures are primarily suitable for clinical use, but also suitable for evaluation. Policymakers could then mandate the integration of standardised, brief self-report measures (e.g., validated scales for parental mental health or confidence) into routine collection points (e.g., universal health visitor checks), which would be used primarily for screening purposes, but also be suitable for researchers to use in evaluation studies.
2. **Researchers to communicate the use of RCD to services.** Many studies noted difficulties with accessing high quality and well completed RCD. We recommend that researchers communicate the impact of RCD findings back to service providers. Demonstrating how this data informs intervention evaluation could improve service delivery, and boost motivation for services to ensure data completeness, accuracy, and accessibility. Further research could explore whether this improves the quality of RCD for evaluating early years interventions.
3. **Researchers to clearly describe RCD sources and how they accessed them.** Descriptions of RCD sources and measures differed between studies, and many studies did not include data availability statements. Clear, consistent descriptions of datasets would enable researchers to better understand the characteristics of different data sources, including their coverage, completeness, and the specific variables captured. Open research practices, such as sharing code used to analyse data and consistent use of relevant reporting guidelines, such as RECORD [78], would enable clearer understanding of data sources used.
4. **Researchers to investigate reasons forunderrepresented outcomes.** Some key outcomes were underutilised, potentially indicating poor data completeness and/or accessibility. For instance, the mandatory 2-year ASQ-3 was not indicated to be used in any evaluation studies, despite child development being a likely outcome for many early years intervention studies. Future work should be dedicated to understanding reasons for their underuse, and potentially developing ways to improve data quality and/or access for these outcomes if required.
5. **Researchers to continue to utilise both RCD and bespoke collected outcomes to understand the effects of interventions.** RCD is currently not sufficient to be used on its own to evaluate most early years interventions and policies. This current review has evidenced that RCD does not capture some key outcomes of early years interventions, and evidenced that many researchers share concerns regarding the quality and completeness of using RCD for research purposes, which is not the primary purpose of RCD. Hence, evaluations with additional bespoke data collection are still required. Data from these studies can be combined with outcomes nested in RCD where possible, to build understanding of the effects of early life interventions.

### Limitations

We did not formally assess the quality or risk of bias of the included studies. Consequently, we could not evaluate the quality of the RCD sources used or the potential impact of data quality on intervention evaluations. Since the use of RCD for research purposes is a rapidly evolving field, these review findings may require updating within a few years. Finally, although we made efforts to include grey literature, sources such as trial registrations and conference abstracts often lacked the detailed methodological information needed to confirm eligibility. This means the current review likely underestimates the volume of relevant studies that exist and may become more widely available in the near future.

### Conclusions

Altogether, this review has highlighted a critical trade-off when using RCD to evaluate interventions: whilst RCD opens up evaluation opportunities, researchers are also limited by what measures are available in RCD. The recommendations call for (1) further research into which outcomes policymakers should integrate into RCD, (2) researchers to communicate findings using RCD to services; (3) researchers to clearly describe data access and sources, (4) researchers to investigate reasons for underrepresented outcomes, and (5) researchers to use both RCD and bespoke collected outcome to understand effects of interventions. With careful considerations, RCD can provide useful insights into the effectiveness of early years interventions, and researchers should continue to use it in combination with other available data. This scoping review reveals an emerging methodology that may have increasing importance for evaluating early years policies and interventions, but may currently be constrained by data availability and quality.

## Data Availability

This scoping review did not include any primary data collection. All data produced in the present study are available upon reasonable request to the authors.
